# Prevalence and Risk Factors of Feline Immunodeficiency Virus and Feline Leukemia Virus Infection in Healthy Cats in Thailand

**DOI:** 10.3389/fvets.2021.764217

**Published:** 2022-01-27

**Authors:** Fabienne Sprißler, Prapaporn Jongwattanapisan, Supol Luengyosluechakul, Rosama Pusoonthornthum, Sven Reese, Michèle Bergmann, Katrin Hartmann

**Affiliations:** ^1^Clinic of Small Animal Medicine, Centre for Clinical Veterinary Medicine, Ludwig-Maximilians-Universität (LMU) Munich, Munich, Germany; ^2^Department of Veterinary Medicine, Chulalongkorn University of Bangkok, Bangkok, Thailand; ^3^Department of Veterinary Sciences, Section for Anatomy, Histology and Embryology, Ludwig-Maximilians-Universität (LMU) Munich, Munich, Germany

**Keywords:** FIV, FeLV, retrovirus infection, ELISA, Bangkok, healthy

## Abstract

Infections with feline immunodeficiency virus (FIV) and feline leukemia virus (FeLV) occur worldwide and are among the most important infectious diseases in cats. The aim of the present study was to determine the prevalence of FIV and FeLV infection in healthy outdoor cats in North, Northeast and Central Thailand. So far, a study on retrovirus prevalence of healthy cats in Thailand in a larger geographic area has not been published yet. In addition, risk factors for FIV and FeLV infections were evaluated. Two hundred sixty healthy cats were prospectively recruited. They originated from 13 locations in North, Northeast, and Central Thailand and were presented for either preventive health care and/or neutering. In each cat, a physical examination was performed to confirm health status. FIV and FeLV status was determined using a commercial rapid enzyme-linked immunosorbent assay (ELISA) (SNAP Combo Plus FeLV/FIV, IDEXX). Risk factors were analyzed by binary logistic regression analysis. Samples of 15/260 (5.8%) cats were positive for FIV antibodies, and 11/260 (4.2%) samples were positive for FeLV antigen. One of the 260 (0.4%) cats was positive for both, FIV and FeLV infection. In binary logistic regression analysis, no parameter was associated with a higher risk for FeLV infection. However, cats had a significantly (*p* = 0.025) higher risk for FIV infection when they were 2 years or older. FIV and FeLV infections occur in healthy cats in North, Northeast and Central Thailand, but prevalence was lower than expected. No risk factors for FeLV infection were detected, but risk for FIV infection increases with age.

## Introduction

Feline immunodeficiency virus (FIV) and feline leukemia virus (FeLV) infections occur in cats worldwide and are associated with several disease syndromes. Prevalence of both infections is highly variable among countries and regions. Prevalence of FIV infection ranges from 2 to 44% worldwide (1–7). In Europe, prevalence is highly variable with up to 30% of cats being infected in countries with large free roaming feline populations such as Italy (8–11). In the US, prevalence ranges from 3% in the overall population to 18% in sick cats (1). In Asia, prevalence varies from 6 to 44% (7, 12–16). FeLV prevalence has been reported in <1 to 31% of cats worldwide, from <1 to 15% in Europe, 2 to 3% in the United States, 3 to 28% in South America and <1 to 24.5% in Asia, Australia and New Zealand (1, 8–11, 14, 15, 17–33). In previous studies, prevalences for FIV and FeLV infection in Asia were higher than in Europe or the United States (1, 13, 14, 16, 17). It was suggested that this might be due to different cat living conditions and vaccination strategies. Cats in Thailand mostly have outdoor access and contact to many other cats of which a high number are strays (16). In 2016, the estimated number of cats in Bangkok was about 4,00,000, of which about 90,000 were strays (32). Free roaming cats are at high risk for retrovirus infections (17, 34). FeLV and FIV vaccines are available in Thailand but are not commonly used.

There is limited information on FIV and FeLV infection prevalence in Thailand, and available studies either included only a low number of cats or investigated cats in only small areas (12, 15, 16, 35–40) (Table 1). Most of these studies looked at cats from Bangkok and its vicinities. In addition, these studies mostly included sick cats (12, 15, 16, 36, 37) (Table 1). So far, there is only one study in a limited region of Northeast Thailand (Khon Kaen city) that evaluated clinically healthy cats in 2018 (40) and only one study investigating risk factors for FIV and FeLV infection in Thailand (15). One recent study looked at risk factors for FeLV only, again in a limited region (Bangkok and Chiang Mai) (35). Thus, the present study aimed to determine the prevalence of FeLV and FIV in healthy outdoor cats in North, Northeast, and Central Thailand. In addition, risk factors for FeLV and FIV infection in Thailand were evaluated.

**Table 1:** Previous studies on prevalence of feline immunodeficiency virus infection, feline leukemia virus infection and double infection in Thailand: year of sampling, number of cats tested, number and percentage of cats positive for feline immunodeficiency virus antibodies and for feline leukemia virus antigen, and references.

**Year**	**Number of tested cats**	**Health status**	**Number of pos. cats (prevalence %)**		**Double infection**	**References**
			**FIV**	**FeLV**
1988	653	not ment.	not det.	6 (0.9)	not ment.	Nilkumhang et al. (36)
1988	110	sick	not det.	23 (20.9)	not ment.	Nilkumhang et al. (36)
1994	145	sick	58 (40.0)	not det.	not ment.	Nilkumhang et al. (37)
1997	28	sick	9 (32.1)	not det.	not ment.	Pusoonthornthum et al. (12)
2003	115	not ment.	6 (5.2)	7 (6.1)	not ment.	Litster et al. (38)
2009	746	sick	150 (20.1)	183 (24.5)	75 (10.1)	Sukhumavasi et al. (15)
2009	133	not ment.	17 (12.8)	19 (14.3)	not ment.	Sattasathuchana et al. (39)
2013/2014	777	sick	42 (5.4)	128 (16.5)	27 (3.5)	Nedumpun et al. (16)
2016	212^a^/216^b^	healthy	13 (6.1)	8 (3.7)	not ment.	Aiyaranoi et al. (40)
**2017**	**260**	**healthy**	**15 (5.8)**	**11 (4.2)**	**1 (0.4)**	**present study**
2017/2018	119	not ment.	not det.	17 (14.3)	not ment.	Capozza et al. (35)

*pos., positive; FIV, feline immunodeficiency virus; FeLV, feline leukemia virus; not ment., not mentioned*.

a *Number of cats tested for FIV infection*.

b *Number of cats tested for FeLV infection*.

## Materials and Methods

### Cats

In total, 260 healthy cats were prospectively recruited from December 2016 to March 2017. Cats originated from 13 different locations (seven in the Bangkok area, six outside of Bangkok) in North, Northeast, and Central Thailand (Supplementary Figure 1) and were presented for either preventive health care and/or neutering. To determine risk factors associated with FIV or FeLV infection, data on environment and history were obtained from owners or caregivers by a questionnaire. Only cats with outdoor access and only cats healthy in physical examination were included.

A minimum sample size of at least 245 cats had been estimated by power analysis, using BIAS for Windows 11.12 (Epsilon, Frankfurt, Germany) based on an assumed infections prevalence of 20% and a maximum 95% CI of 10 percentage points. Animal Ethics committee approval of the Faculty of Veterinary Science, Chulalongkorn University of Bangkok, Thailand (approval number: 1731042) and informed consent of cat owners were obtained.

Only cats were included that had not been vaccinated against FIV or FeLV (139 of the cats had received other vaccines, primarily rabies).

All samples were taken by the corresponding author (FS) either at the Chulalongkorn University Veterinary Clinic, in a shelter in Bangkok, or during several (neutering) programs. Blood was taken either from the jugular or cephalic vein. Serum samples were frozen and stored at −20°C. FIV and FeLV tests were performed by the corresponding author (FS) after all samples were collected.

### Retrovirus Testing

Cats were tested for FIV antibodies and FeLV antigen using a commercial rapid enzyme-linked immunosorbent assay (ELISA) (SNAP Combo Plus FeLV/FIV, IDEXX GmbH, Ludwigsburg, Germany).

### Risk Factor Analysis

To determine risk factors (Table 2) associated with FIV and FeLV infection, data on characteristics (gender, age, reproduction status), location and month of sampling, reason for presentation (either neutering or general health check), origin (domestic/feral), environment (urban/rural) and contact with other cats were obtained from the owners or the caregiver by a questionnaire.

**Table 2:** Risk factors of cats with and without feline immunodeficiency virus and feline leukemia virus infection and binary logistic regression analysis.

		***n***	**Number of FIV-antibody-positive cats (%)**	**Logistic regression analysis *p***	**Number of FeLV-antigen-positive cats (%)**	**Logistic regression analysis *p***
**Risk factors**	**categories**	260	15 (5.8)	-	11 (4.2)	-
age (*n* = 233)	≥ 2 years	98	9 (9.2)	0.025*	7 (7.1)	0.052
	<2 years	135	3 (2.2)		1 (2.2)	
gender (*n* = 250)	male	113	7 (6.2)	0.961	6 (5.3)	0.362
	female	137	7 (5.1)		4 (2.9)	
reproduction status (*n* = 256)	neutered	19	1 (5.3)	0.554	0 (0.0)	0.998
	intact	237	13 (5.5)		11 (4.4)	
origin (*n* = 255)	urban	207	11 (5.3)	0.181	8 (3.9)	0.970
	rural	48	3 (6.3)		3 (6.3)	
part of Thailand (*n* = 260)	Central vs. North	163 vs 18	8 (4.9) vs. 2 (11.1)	0.999	5 (3.1) vs. 6 (33.3)	0.999
	Central vs. Northeast	163 vs 79	8 (4.9) vs. 5 (6.3)	0.572	5 (3.1) vs. 0 (0.0)	0.997
reason for presentation (*n* = 255)	general health check	14	0 (0.0)	0.998	0 (0.0)	0.998
	neutering	241	15 (6.2)		11 (4.6)	
origin (*n* = 256)	privately owned	247	13 (5.3)	0.999	11 (4.5)	1.000
	feral	9	1 (11.1)		0 (0.0)	
contact with other cats (*n* = 256)	domestic and feral vs. feral	212 vs. 5	10 (4.7) vs. 1 (20.0)	0.999	4 (1.9) vs. 0 (0.0)	1.000
	domestic and feral vs. domestic	212 vs. 37	10 (4.7) vs. 3 (8.1)	0.999	4 (1.9) vs. 7 (18.9)	0.999
	domestic and feral vs. no contact	212 vs. 2	10 (4.7) vs. 0 (0.0)	1.000	4 (1.9) vs. 0 (0.0)	1.000
month of sampling (*n* = 260)	Feb vs. Mar	122 vs. 94	5 (4.1) vs. 5 (5.3)	0.378	2 (1.6) vs. 2 (2.1)	0.378
	Feb vs. Dec	122 vs. 12	5 (4.1) vs. 3 (25.0)	0.999	2 (1.6) vs. 1 (8.3)	0.999
	Feb vs. Jan	122 vs. 32	5 (4.1) vs. 2 (6.3)	1.000	2 (1.6) vs. 6 (18.8)	1.000

n, numbers of cats in every group; FIV, feline immunodeficiency virus; FeLV, feline leukemia virus; p, p-value; -, calculation due to no comparable group not available; * = significant, OR 4.958, 95% CI 1.255–20.074; vs., versus; Jan, January; Feb, February; Mar, March; Dec, December; total number of cats for each parameter ranged from 233 to 260 because not every parameter was specified by the owners/caretakers.

### Statistical Analysis

Statistical analysis was performed using SPSS 28.0. Prevalence of FIV and FeLV infection and its 95% CI were calculated. A binary logistic regression analysis was performed for risk factor analysis. A *p*-value < 0.05 was considered as statistically significant.

## Results

### Study Population

All cats were domestic short hair (DSH) and ranged in age from 6 months to 10 years (median age: 1.8 years). One hundred thirteen male and 137 female cats were included (Table 2). In 10 cats, sex was not recorded.

### Prevalence of FIV and FeLV

Samples of 15/260 cats (5.8%; 95% CI: 3.1–8.5%) were positive for FIV antibodies. Samples of 11/260 cats (4.2%; 95% CI: 1.9–6.9%) were positive for FeLV antigen. One of 260 cats (0.4%; 95% CI: 0.0–2.1%) was positive for both, FIV and FeLV infection. This cat was sampled in urban Ban Mot, Bangkok. It was a privately owned, male, intact cat of unknown age. Table 3 and Supplementary Figure 1 shows sample locations, number of cats sampled in each location, and geographical distribution of FIV- and FeLV-infected cats.

**Table 3:** Sample locations, region, environment, number of cats sampled and number and percentage of cats with feline immunodeficiency virus and feline leukemia virus infection.

**Sample location**	**Region**	**Environment**	**Number of cats sampled**	**FIV infection (%)**	**FeLV infection (%)**
Lamphun	N	rural	18	2 (11.1)	6 (33.3)
Amnat Charoen	NO	rural	7	2 (28.6)	0 (0)
Nakhon Ratchasima	NO	urban and rural	49	1 (2.0)	0 (0)
Udon Thani	NO	urban	23	2 (8.7)	0 (0)
Bangkok	C	urban	138	8 (5.8)	5 (3.6)
Nakhon Pathom	C	urban	14	0 (0)	0 (0)
Ratchaburi	C	urban	11	0 (0)	0 (0)

*N, north; NO, northeast; C, central; FIV, feline immunodeficiency virus; FeLV, feline leukemia virus. The environment factor (urban/rural) was determined by the location of the sampling and not by information of the owners/caretakers. All 260 cats were included*.

### Risk Factors

Using binary logistic regression analysis, several risk factors were evaluated (Table 2). The probability to be infected with FIV was significantly (*p* = 0.025) higher for cats at the age of 2 years or older (Table 2). No factor was significantly associated with FeLV infection.

## Discussion

The present study investigated the prevalence of FIV and FeLV in 260 clinically healthy cats in North, Northeast, and Central Thailand. A FIV prevalence of 4.2% (11/260) was detected. Previous studies only investigated cats from Bangkok and its vicinities for FIV infection, and prevalence of up to 40.1% was described (12, 15, 16, 37, 38, 40) (Table 1). Prevalence of FIV infection in the present study was much lower than in most previous reports, potentially due to different study populations, such as concerning health status, sample locations, and living conditions. In the present study, healthy cats in a larger region were included whereas in earlier studies, cats were presented to different veterinary clinics with clinical signs in Thailand (12, 15, 16, 37, 38) (Table 1). A significantly higher prevalence for FIV infection in sick cats was determined in studies in other countries before which might explain the difference (14, 37, 41). In a recent study, Aiyaranoi et al. (2018) investigated cats from Khon Kaen city, which is located in Northeast of Thailand and reported an prevalence of 6.1% (40). The cat populations of the present study and of the study in Khon Kaen were very similar and comparable regarding age and gender. Furthermore, both studies included only healthy cats. In the present study, a prevalence of 6.3% was determined for cats originating from Northeast Thailand, and this is indeed very similar to that of the study of Ayaranoi et al. (40). It should be considered that in the present study, in the region of Amnat Charoen 28.6% (2/7) of sampled cats tested positive, whereas in Nakhon Ratschasima and Udonthani lower prevalences were detected (Table 3). Amnat Charoen is one of the poorest provinces in Thailand and situated at the border to Laos (42). There are no studies available about FIV prevalence in Laos, but high FIV prevalences in cats in Laos could be one explanation for the very high prevalence in Amnat Charoen (15). FIV prevalence of 4.9% was determined in Bangkok and its vicinities in the present study. In contrast, a very high prevalence (40.1%) of FIV infection was detected in the same area in 1994 (38). In a study from 1997, prevalence of FIV infection was 32.1% (12), in 2009, prevalence was 20.1% (15), and a recent study determined a prevalence of 5.4% for cats in Bangkok and its vicinities (16), which is similar to the present study. Therefore, prevalence has considerably decreased in the last 25 years (Table 1). Nowadays, there are several private and governmental organizations that neuter cats in the Bangkok area. As a result there are less intact fighting male cats in this area and transmission of FIV could be reduced (16).

In the binary logistic regression analysis of the present study, cats at the age of 2 years or older had a higher risk to be FIV-infected. This is in accordance with other studies performed in Thailand and other countries worldwide (9, 10, 12, 14, 15, 29, 41, 43), and can be explained by an increasing risk of exposure in older cats. No other risk factor was found to be significantly associated with a higher risk for FIV infection, although in other studies male intact cats were at higher risk to be infected with FIV (10, 15, 29, 41).

In the present study, the FeLV prevalence was 4.2% in North, Northeast, and Central Thailand. So far, there is only one study published on FeLV prevalence with a small study population (119 cats) from Bangkok and Chiang Mai, which is situated in the North of Thailand (35). The authors determined a prevalence of progressive FeLV infection of 14.3 % (35). The number of cats sampled in Chiang Mai was neither specified in this study (35), nor in an overlapping larger study in which cats and dogs from Eastern and Southeast Asia were tested for vector-borne pathogens and ectoparasites (44). There is no study that investigated FeLV prevalence in Northeast Thailand so far. Previous studies in Bangkok and its vicinities mostly included sick cats and even recent studies detected prevalences up to 24.5% (15, 16, 37). One study found a prevalence of 20.9% in sick cats (37) (Table 1). The previous study conducted in Bangkok and Chiang Mai included not only, but also sick cats as the study involved cats with clinical signs (i.e., enlarged lymph nodes and skin abnormalities) from academic institutions and private facilities (44). This could be one explanation for the higher prevalence determined in that study. In one study from 2016 in which only healthy cats were included, prevalence of progressive FeLV infection (4.2%) was comparable to the prevalence determined in the present study (40).

In the present study, only healthy cats were included and this might explain the much lower prevalence when compared to most previous studies. Being ill has been detected as a significant risk factor for FeLV infection in several studies worldwide (45). All FeLV-infected cats in the present study originated either from Bangkok or Lamphun, a city in North Thailand (Table 3). In the other five sample locations, no cat tested positive. Possibly, FeLV is endemic in Bangkok and Lamphun in contrast to other locations. The observed differences might be attributed to variation among geographic regions, cat population densities, lifestyles and control policies and practices among different regions. Fromont et al. (2003) investigated models to predict dynamics of FeLV in cat populations and found that extinction of FeLV is possible in small populations (46). Furthermore, these models showed that FeLV dynamics depend on size of the population and the relationship between host density and the pattern of contact of individual cats (46). Nakumara et al. (2000) even suggested that some cat populations in Asia might be free of FeLV infection; as an example, none of the tested cats in North and South Vietnam was progressively infected with FeLV (13).

The higher prevalence of FeLV in the study of Capozza et al. (2021) could be explained by a higher regional prevalence in Bangkok and North Thailand (where Chiang Mai is situated) which is in accordance to the present study (35). Capozza et al. (2021) found a significant (*p* < 0.05) association between Thailand as country of cats' origin, adult age, abnormal oral mucosa, and positive FeLV-antigen results (35). In the present study, adult age was not a significant risk factor (*p* = 0.052) for FeLV infection. As only healthy cats were included, clinical sings could not be evaluated as a potential risk factor.

One limitation of the study is that samples were only tested for FeLV antigen and thus, only cats with progressive infection (and occasionally those with early regressive infection) were detected but most regressively infected and all abortively infected cats were likely missed (47). After a short period of viremia, regressively infected cats only harbor provirus in bone marrow and blood cells, and thus, true FeLV prevalence is always higher than the prevalence of antigen-positive cats. Furthermore, confirmatory testing was not performed and therefore, potentially false positive results cannot be completely ruled out. Especially FeLV polymerase chain reaction would have been very interesting not only as confirmatory test but also to detect regressive FeLV infection. These additional tests should be investigated in further studies.

## Conclusion

FIV prevalence of 5.8% and FeLV infection prevalence of 4.2% were detected in healthy cats in North, Northeast, and Central Thailand. Cats had a significantly *(p* = 0.025) higher risk for FIV infection when they were 2 years or older. This study can help to monitor FIV and FeLV infections in cats in Thailand and develop control strategies such as recommendation for vaccination. Cats in the present study were not vaccinated against FIV and/or FeLV. Both vaccines are available in Thailand, but are not widely used. FIV vaccination is discussed controversially, according to the present study, FeLV infection is still endemic in Thailand although prevalence is decreasing, and therefore, vaccination against FeLV is highly recommended.

## Data Availability Statement

The raw data supporting the conclusions of this article will be made available by the authors, without undue reservation.

## Ethics Statement

The animal study was reviewed and approved by Animal Ethics Committee of the Faculty of Veterinary Science, Chulalongkorn University of Bangkok, Thailand (approval number: 1731042). Written informed consent was obtained from the owners for the participation of their animals in this study.

## Author Contributions

KH, FS, and MB contributed to conception and design of the study. FS performed the sampling and testing with the help of SL, PJ, and RP. FS organized the data base and wrote the first draft of the manuscript. SR performed the statistical analysis. KH and MB did the proof-reading. All authors contributed to manuscript revision, read, and approved the submitted version.

## Funding

This research was partially funded by MSD Animal Health, the Netherlands. MSD Animal Health played no role in the collection and interpretation of data, or in the decision to submit the manuscript for publication.

## Conflict of Interest

The authors declare that the research was conducted in the absence of any commercial or financial relationships that could be construed as a potential conflict of interest.

## Publisher's Note

All claims expressed in this article are solely those of the authors and do not necessarily represent those of their affiliated organizations, or those of the publisher, the editors and the reviewers. Any product that may be evaluated in this article, or claim that may be made by its manufacturer, is not guaranteed or endorsed by the publisher.

## References

[1] Levy JK Scott HM Lachtara JL Crawford PC. Seroprevalence of feline leukemia virus and feline immunodeficiency virus infection among cats in North America and risk factors for seropositivity. J Am Vet Med Assoc. (2006) 228:371–6. 10.2460/javma.228.3.371 16448357

[2] Little S Sears W Lachtara J Bienzle D. Seroprevalence of feline leukemia virus and feline immunodeficiency virus infection among cats in Canada. Can Vet J. (2009) 50:644–8. 19721785 PMC2684053

[3] Little SE. Feline immunodeficiency virus testing in stray, feral, and client-owned cats of Ottawa. Can Vet J. (2005) 46:898–901. 16454381 PMC1255591

[4] Burling AN Levy JK Scott HM Crandall MM Tucker SJ Wood EG. Seroprevalences of feline leukemia virus and feline immunodeficiency virus infection in cats in the United States and Canada and risk factors for seropositivity. J Am Vet Med Assoc. (2017) 251:187–94. 10.2460/javma.251.2.187 28671491

[5] Szilasi A Dénes L Krikó E Heenemann K Ertl R Mándoki M. Prevalence of feline immunodeficiency virus and feline leukaemia virus in domestic cats in Hungary. JFMS Open Rep. (2019) 5:1–7. 10.1177/2055116919892094 31839979 PMC6904780

[6] Sacristán I Sieg M Acuña F Aguilar E García S López MJ. Molecular and serological survey of carnivore pathogens in free-roaming domestic cats of rural communities in southern Chile. J Vet Med Sci. (2019) 81:1740–8. 10.1292/jvms.19-0208 31611482 PMC6943315

[7] Ishida T Washizu T Toriyabe K Motoyoshi S Tomoda I Pedersen NC. Feline immunodeficiency virus infection in cats of Japan. J Am Vet Med Assoc. (1989) 194:221–5. 2537270

[8] Dorny P Speybroeck N Verstraete S Baeke M De Becker A Berkvens D. Serological survey of *Toxoplasma gondii*, feline immunodeficiency virus and feline leukaemia virus in urban stray cats in Belgium. Vet Rec. (2002) 151:626–9. 10.1136/vr.151.21.626 12479298

[9] Bandecchi P. Dell'Omodarme M, Magi M, Palamidessi A, Prati MC. Feline leukaemia virus (FeLV) and feline immunodeficiency virus infections in cats in the Pisa district of Tuscany, and attempts to control FeLV infection in a colony of domestic cats by vaccination. Vet Rec. (2006) 158:555–7. 10.1136/vr.158.16.555 16632529

[10] Spada E Proverbio D. della Pepa A Perego R Baggiani L DeGiorgi GB. Seroprevalence of feline immunodeficiency virus, feline leukaemia virus and Toxoplasma gondii in stray cat colonies in northern Italy and correlation with clinical and laboratory data. J Feline Med Surg. (2012) 14:369–77. 10.1177/1098612X12437352 22318850 PMC10822588

[11] Rypula K Ploneczka-Janeczko K Bierowiec K Kumala A Sapikowski G. Prevalence of viral infections in cats in southwestern Poland in the years 2006 to 2010. Berl Munch Tierarztl Wochenschr. (2014) 127:163–5. 24693663

[12] Pusoonthornthum R Oraveerakul K Uthaichotiwan P Cheusiri K. Prevalence of feline immunodeficiency virus (FIV) in cats with chronic diseases. Thai J Vet Med. (1998) 28:77–8.

[13] Nakamura K Miyazawa T Ikeda Y Sato E Nishimura Y Takahashi E. Contrastive prevalence of feline retrovirus infections between northern and southern Vietnam. J Vet Med Sci. (2000) 62:921–3. 10.1292/jvms.62.921 10993195

[14] Bande F Arshad SS Hassan L Zakaria Z Sapian NA Rahman NA. Prevalence and risk factors of feline leukaemia virus and feline immunodeficiency virus in peninsular Malaysia. BMC Vet Res. (2012) 8:33. 10.1186/1746-6148-8-33 22439903 PMC3349470

[15] Sukhumavasi W Bellosa ML Lucio-Forster A Liotta JL Lee AC Pornmingmas P. Serological survey of *Toxoplasma gondii, Dirofilaria immitis*, feline immunodeficiency virus (FIV) and feline leukemia virus (FeLV) infections in pet cats in Bangkok and vicinities, Thailand. Vet Parasitol. (2012) 188:25–30. 10.1016/j.vetpar.2012.02.021 22497870

[16] Nedumpun T Piamsomboon P Chanchaithong P Taweethavonsawat P Chungpivat S Suradhat S. Prevalence and distributions of feline immunodeficiency virus and feline leukemia virus infections in Bangkok and its vicinity, Thailand during 2013-2014. Thai J Vet Med. (2015) 45:449–53.

[17] Arjona A Escolar E Soto I Barquero N Martin D Gomez-Lucia E. Seroepidemiological survey of infection by feline leukemia virus and immunodeficiency virus in Madrid and correlation with some clinical aspects. J Clin Microbiol. (2000) 38:3448–9. 10.1128/JCM.38.9.3448-3449.2000 10970400 PMC87403

[18] Gleich SE Krieger S Hartmann K. Prevalence of feline immunodeficiency virus and feline leukaemia virus among client-owned cats and risk factors for infection in Germany. J Feline Med Surg. (2009) 11:985–92. 10.1016/j.jfms.2009.05.019 19616984 PMC11318771

[19] Hellard E Fouchet D Santin-Janin H Tarin B Badol V Coupier C. When cats' ways of life interact with their viruses: a study in 15 natural populations of owned and unowned cats (*Felis silvestris catus*). Prev Vet Med. (2011) 101:250–64. 10.1016/j.prevetmed.2011.04.020 21705099

[20] Englert T Lutz H Sauter-Louis C Hartmann K. Survey of the feline leukemia virus infection status of cats in Southern Germany. J Feline Med Surg. (2012) 14:392–8. 10.1177/1098612X12440531 22403413 PMC10822583

[21] Bande F Arshad SS Hassan L Zakaria Z. Molecular detection, phylogenetic analysis, and identification of transcription motifs in feline leukemia virus from naturally infected cats in Malaysia. Vet Med Int. (2014) 2014:760961. 10.1155/2014/760961 25506469 PMC4251355

[22] Najafi H Madadgar O Jamshidi S Ghalyanchi Langeroudi A Darzi Lemraski M. Molecular and clinical study on prevalence of feline herpesvirus type 1 and calicivirus in correlation with feline leukemia and immunodeficiency viruses. Vet Res Forum. (2014) 5:255–61. 25610576 PMC4299990

[23] Cong W Meng QF Blaga R Villena I Zhu XQ Qian AD. *Toxoplasma gondii, Dirofilaria immitis*, feline immunodeficiency virus (FIV), and feline leukemia virus (FeLV) infections in stray and pet cats (*Felis catus*) in northwest China: co-infections and risk factors. Parasitol Res. (2016) 115:217–23. 10.1007/s00436-015-4738-y 26362646

[24] Garigliany M Jolly S Dive M Bayrou C Berthemin S Robin P. Risk factors and effect of selective removal on retroviral infections prevalence in Belgian stray cats. Vet Rec. (2016) 178:45. 10.1136/vr.103314 26744011

[25] Hwang J Gottdenker N Min MS Lee H Chun MS. Evaluation of biochemical and haematological parameters and prevalence of selected pathogens in feral cats from urban and rural habitats in South Korea. J Feline Med Surg. (2016) 18:443–51. 10.1177/1098612X15587572 26018551 PMC11185230

[26] Westman M Norris J Malik R Hofmann-Lehmann R Harvey A McLuckie A. The diagnosis of feline leukaemia virus (FeLV) Infection in owned and group-housed rescue cats in Australia. Viruses. (2019) 11:503. 10.20944/preprints201904.0278.v1 31159230 PMC6630418

[27] Biezus G Machado G Ferian PE da Costa UM Pereira L Withoeft JA. Prevalence of and factors associated with feline leukemia virus (FeLV) and feline immunodeficiency virus (FIV) in cats of the state of Santa Catarina, Brazil. Comp Immunol Microbiol Infect Dis. (2019) 63:17–21. 10.1016/j.cimid.2018.12.004 30961813

[28] Luckman C Gates MC. Epidemiology and clinical outcomes of feline immunodeficiency virus and feline leukaemia virus in client-owned cats in New Zealand. JFMS Open Rep. (2017) 3:2055116917729311. 10.1177/2055116917729311 30202540 PMC6125856

[29] Sivagurunathan A Atwa AM Lobetti R. Prevalence of feline immunodeficiency virus and feline leukaemia virus infection in Malaysia: a retrospective study. JFMS Open Rep. (2018) 4:1–5. 10.1177/2055116917752587 29568541 PMC5858631

[30] Hofmann-Lehmann R Gonczi E Riond B Meli M Willi B Howard J. Feline leukemia virus infection: importance and current situation in Switzerland. Schweiz Arch Tierheilkd. (2018) 160:95–105. 10.17236/sat00146 29386166

[31] Lacerda LC Silva AN Freitas JS Cruz RDS Said RA Munhoz AD. Feline immunodeficiency virus and feline leukemia virus: frequency and associated factors in cats in northeastern Brazil. Genet Mol Res. (2017) 16:1–8. 10.4238/gmr16029633 28510253

[32] Department Department of Livestock Development BoDCaVS Thailand. Survey of Dog and Cat, Rabies Campaign. (2016). Available online at: http://dcontrol.dld.go.th/dcontrol/index.php/rabies/747-dogpop2016 (accessed April 4, 2020).

[33] Hoover EA Mullins JI. Feline leukemia virus infection and diseases. J Am Vet Med Assoc. (1991) 199:1287–97. 1666070

[34] Muirden A. Prevalence of feline leukaemia virus and antibodies to feline immunodeficiency virus and feline coronavirus in stray cats sent to an RSPCA hospital. Vet Rec. (2002) 150:621–5. 10.1136/vr.150.20.621 12046785

[35] Capozza P Lorusso E Colella V Thibault J Tan D Tronel J. Feline leukemia virus in owned cats in Southeast Asia and Taiwan. Vet Microbiol. (2021) 254:109008. 10.1016/j.vetmic.2021.109008 33582484

[36] Nilkumhang P Tipsawake S Srisuparb K. An epidemiological survey of cats for evidence of feline leukemia virus antigen by ELISA test. J Thai Vet Med Assoc. (1988) 39:73–7.

[37] Nilkumhang P Theeraleekul T Silpabhadung U. Feline immunodeficiency virus infection: prevalence and clinical findings. J Thai Vet Pract. (1994) 6:11–22.23734699

[38] Litster A Nilkumhang P. Prevalence of feline leukemia virus and feline immunodeficiency virus infection in Thailand [Conference presentation]. In: The 28th World Congress of the World Small Animal Veterinary Association. Bangkok (2003) p. 714

[39] Sattasathuchana P Puripanpipat S Sreesampan S Nilkhamhang P. The study of feline leukemia virus and feline immunodeficiency virus infection rate in Bangkok and its vicinities [Conference presentation]. In: Regional Veterinary Congress of Veterinary Practitioner Association of Thailand. (2009) Bangkok, p. 339–41

[40] Aiyaranoi K Boonchalaew N Chawnan N Chotiku S Kampa J. Prevalence of feline immunodeficiency virus & feline leukemia virus in clinically healthy cats in Khon Kaen province. Thai J Vet Med. (2018) 48:117–21.

[41] Chhetri BK Berke O Pearl DL Bienzle D. Comparison of risk factors for seropositivity to feline immunodeficiency virus and feline leukemia virus among cats: a case-case study. BMC Vet Res. (2015) 11:30. 10.1186/s12917-015-0339-3 25889006 PMC4332748

[42] Healy AJ Jitsuchon S. Finding the poor in Thailand. J Asian Econ. (2007) 18:739–59. 10.1016/j.asieco.2007.05.004

[43] Hitt ME Spangler L McCarville C. Prevalence of feline immunodeficiency virus in submissions of feline serum to a diagnostic laboratory in Atlantic Canada. Can Vet J. (1992) 33:723–6. 17424114 PMC1481433

[44] Colella V Nguyen VL Tan DY Lu N Fang F Zhijuan Y. Zoonotic vectorborne pathogens and ectoparasites of dogs and cats in Eastern and Southeast Asia. Emerg Infect Dis. (2020) 26:1221. 10.3201/eid2606.191832 32441628 PMC7258489

[45] Galdo Novo S Bucafusco D Diaz LM Bratanich AC. Viral diagnostic criteria for feline immunodeficiency virus and feline leukemia virus infections in domestic cats from Buenos Aires, Argentina. Rev Argent Microbiol. (2016) 48:293–7. 10.1016/j.ram.2016.07.003 27825735

[46] Fromont E Pontier D Langlais M. Disease propagation in connected host populations with density-dependent dynamics: the case of the feline leukemia virus. J Theor Biol. (2003) 223:465–75. 10.1016/S0022-5193(03)00122-X 12875824

[47] Hofmann-Lehmann R Huder JB Gruber S Boretti F Sigrist B Lutz H. Feline leukaemia provirus load during the course of experimental infection and in naturally infected cats. J Gen Virol. (2001) 82(Pt 7):1589–96. 10.1099/0022-1317-82-7-1589 11413369

