# Supplementary figures and images for: Prevalence and Risk Factors of Feline Immunodeficiency Virus and Feline Leukemia Virus Infection in Healthy Cats in Thailand
Source: Front Vet Sci. 2022 Jan 27;8:764217. doi: 10.3389/fvets.2021.764217 (PMC8862143; doi:10.3389/fvets.2021.764217)

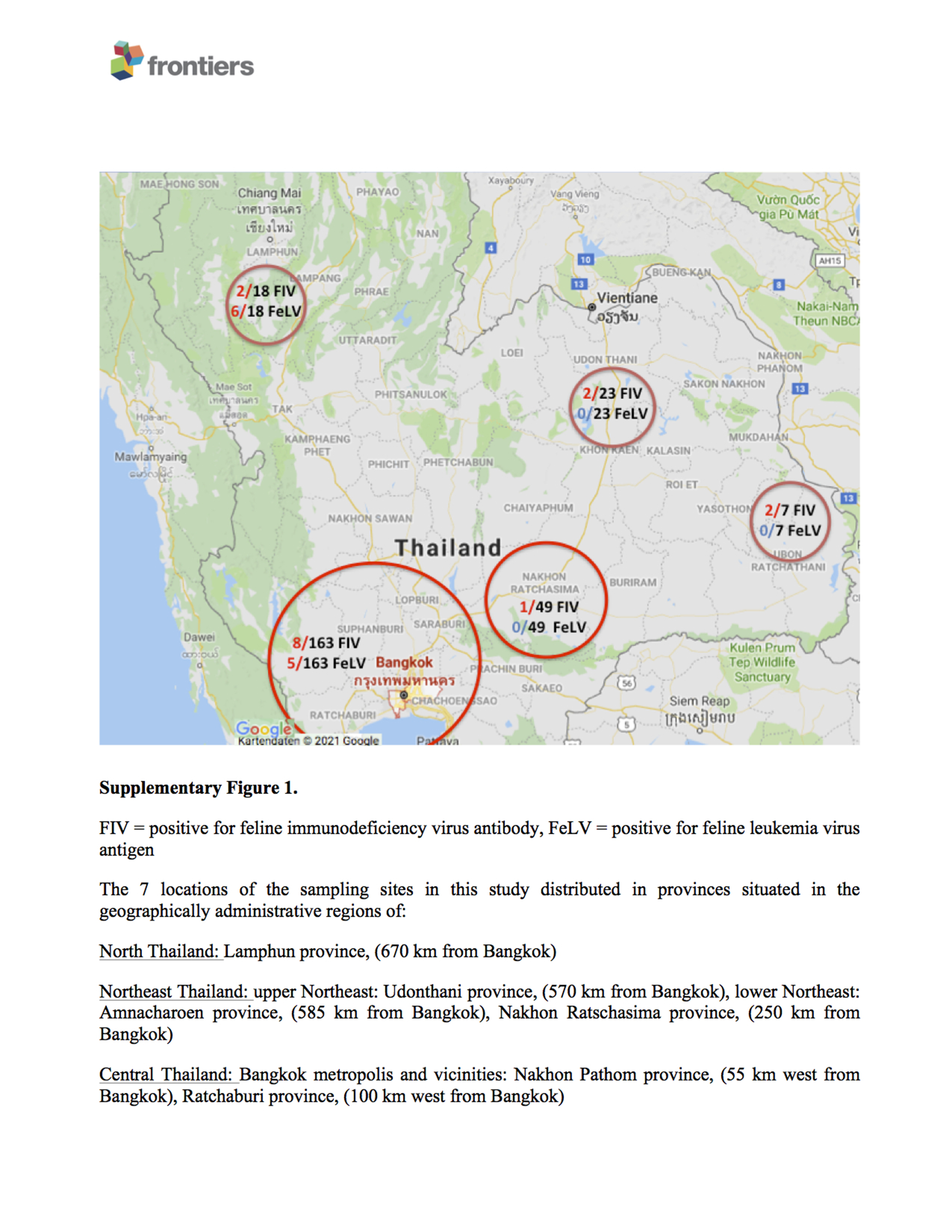

Supplement: Supplementary file 1 [file Image_1.jpg]
